# Renal oncocytoma characterized by the defective complex I of the respiratory chain boosts the synthesis of the ROS scavenger glutathione

**DOI:** 10.18632/oncotarget.22413

**Published:** 2017-11-11

**Authors:** Gerrit Kürschner, Qingzhou Zhang, Rosanna Clima, Yi Xiao, Jonas Felix Busch, Ergin Kilic, Klaus Jung, Nikolaus Berndt, Sascha Bulik, Hermann-Georg Holzhütter, Giuseppe Gasparre, Marcella Attimonelli, Mohan Babu, David Meierhofer

**Affiliations:** ^1^ Max Planck Institute for Molecular Genetics, Mass Spectrometry Facility, Berlin, Germany; ^2^ Technical University of Berlin, Institute of Bioanalytics, Department of Biotechnology, Berlin, Germany; ^3^ University of Regina, Department of Biochemistry, Regina, Canada; ^4^ University of Bari, Department of Biosciences, Biotechnology and Biopharmaceutics, Bari, Italy; ^5^ Department of Medical and Surgical Sciences-DIMEC, Medical Genetics Unit, University of Bologna, Bologna, Italy; ^6^ Freie Universität Berlin, Fachbereich Biologie, Chemie, Pharmazie, Berlin, Germany; ^7^ University Hospital Charité, Department of Urology, Berlin, Germany; ^8^ University Hospital Charité, Institute of Pathology, Berlin, Germany; ^9^ Berlin Institute for Urologic Research, Berlin, Germany; ^10^ Charité University Medicine Berlin, Institute of Biochemistry Computational Systems Biochemistry Group, Berlin, Germany

**Keywords:** renal oncocytoma, complex I deficiency, glutathione metabolism, mtDNA mutation

## Abstract

Renal oncocytomas are rare benign tumors of the kidney and characterized by a deficient complex I (CI) enzyme activity of the oxidative phosphorylation (OXPHOS) system caused by mitochondrial DNA (mtDNA) mutations. Yet, little is known about the underlying molecular mechanisms and alterations of metabolic pathways in this tumor. We compared renal oncocytomas with adjacent matched normal kidney tissues on a global scale by multi-omics approaches, including whole exome sequencing (WES), proteomics, metabolomics, and metabolic pathway simulation. The abundance of proteins localized to mitochondria increased more than 2-fold, the only exception was a strong decrease in the abundance for CI subunits that revealed several pathogenic heteroplasmic mtDNA mutations by WES. We also observed renal oncocytomas to dysregulate main metabolic pathways, shunting away from gluconeogenesis and lipid metabolism. Nevertheless, the abundance of energy carrier molecules such as NAD^+^, NADH, NADP, ATP, and ADP were significantly higher in renal oncocytomas. Finally, a substantial 5000-fold increase of the reactive oxygen species scavenger glutathione can be regarded as a new hallmark of renal oncocytoma. Our findings demonstrate that renal oncocytomas undergo a metabolic switch to eliminate ATP consuming processes to ensure a sufficient energy supply for the tumor.

## INTRODUCTION

Renal oncocytomas classified as benign renal epithelial neoplasms [1, 2] that are derived from intercalated cells comprise only a small subset (3% to 9%) of all primary renal neoplasms, and can be cured by nephrectomy. The hallmark of renal oncocytomas is the accumulation of mitochondria [3] and the highly diminished or complete loss of complex I (CI) enzyme activity within the electron transport chain [4, 5]. CI deficiency is mainly due to mutations in mitochondrial DNA (mtDNA), particularly, but not exclusively, in CI genes [4, 6]. Most mtDNA mutations detected in renal oncocytomas are well above the threshold for a pathogenic phenotype. The level of heteroplasmy can vary between cells in the same tissue or organ, and the proportion of mutant mtDNA determines the penetrance and severity of disease expression [7]. Nonetheless, why and how mutated mtDNA specifically accumulates in oncocytomas still remains unclear.

Most mtDNA mutations in renal oncocytomas occur in homopolymeric G-C and A-T stretches [4], and defects in mtDNA replication (or lack of an efficient repair system) may induce randomly such genetic lesions during cancer progression. Low-heteroplasmy mutations may confer a selection advantage due to an increase in reactive oxygen species (ROS) levels, but when shifted to homoplasmy, mtDNA mutations appear to reduce tumor growth due to their effect on respiratory complex assembly [8, 9]. This would lead to oncocytic transformation, conferring, in most cases, an indolent, low-proliferating, non-invasive behavior. Most notably, the main source of ROS are CI and CIII of the respiratory chain [10, 11]. CI-dependent increase of ROS levels was shown via the NDUFS1 subunit, with the loss of NADH dehydrogenase activity as a caspase-3 target [12], or via a caspase independent pathway, where granzyme A (GZMA) cleaves the CI subunit NDUFS3 [13].

Hypoxia-inducible factor 1-alpha (HIF1A) is a master transcriptional regulator of the adaptive response to hypoxia, and more than 100 genes involved in glucose transporters, glycolytic enzymes, and vascular endothelial growth factor are known to be activated upon its stabilization [14]. Strikingly, however, oncocytic tumors are non-responsive to hypoxia due to HIF1A chronic destabilization [15]. Since mtDNA mutations in CI that cause deficient CI enzyme activity and increased mitochondrial mass are characteristics of renal oncocytomas. The latter serve as an ideal tool to study the relationship between mtDNA mutations of the oxidative phosphorylation (OXPHOS) system and an indolent cancer phenotype. Therefore, we attempted to investigate which metabolic pathways are altered in this chronic metabolic deficiency as a consequence of compromised mitochondrial respiration in renal oncocytomas on a global scale.

To fill in the aforesaid knowledge gaps, we undertook a multi-‘omics’ survey to compare renal oncocytomas and patient-matched adjacent healthy kidney tissues. Whole exome sequencing (WES) led to the identification of cancer-specific mtDNA mutations that affect protein function. The proteome survey identified increased mitochondrial mass, except for CI subunits in renal oncocytomas. This was in contrast to transcriptome data, where CI genes were increased [16], leading to the conclusion that the low level heteroplasmic mtDNA mutations are causative for disassembled and nonfunctional CI. Surprisingly, pathways which could compensate defective aerobic respiration, such as the pentose phosphate pathway was significantly diminished. Likewise, the gluconeogenetic enzyme fructose-1,6-bisphosphatase 1 (FBP1), which was previously shown to oppose renal cell carcinoma (ccRCC) progression [17] was more than 200-fold decreased in renal oncocytomas. However, our most striking finding was the tremendous increase of the ROS scavenger glutathione (GSH) that could have been triggered due to the consequence of high ROS production owing to defective respiration, and can be regarded as a new metabolic hallmark in renal oncocytomas. Thus, stalled glycolytic flux with defective mitochondrial respiration and high GSH levels might determine the benign and dormant fate of this tumor and oppose progression to malignant states.

## RESULTS

### WES analysis indicates increased number of nuclear mutations in renal oncocytomas

To gain insight on the molecular cause of the CI enzyme activity decrease in renal oncocytomas [4, 5], we performed WES of renal oncocytomas and their matching healthy kidney tissues with the aim to isolate all somatic mutation signatures showing 200X minimum sequencing coverage across the targeted bases, and minimum read depths of 61.21X (Supplementary Table 1). In total, we identified 1597 nuclear sequence variations, of which 799 were silent, 760 missense, 19 nonsense, 11 frame-shift deletions, and 8 frame-shift insertion mutations. The number of tumor-specific nuclear mutations (except silent ones) in oncocytomas were in the range of 65 to 329 (median of 78) per renal oncocytoma (Supplementary Table 2). Majority of the nuclear point mutation spectrum includes C>A (400), C>G (426), C>T (1696), T>A (259), T>C (1228), and T>G (274). With respect to previous observation [16], the frequency of transition in our European patient cohort with renal oncocytomas tested was 2-fold higher than that of transversions, whereas C>T (*p* = 1.03 × 10^−6^) and T>C (*p* = 2.02 × 10^−2^) were significantly enriched in oncocytomas (Supplementary Figure 1, Supplementary Table 3). While the “mutational signature” C>T has been found in all cancer types and is the resultant of an endogenous mutational process initiated by spontaneous deamination of 5-methylcytosine [18], the correlation of C>T transitions with age [19] is indicative of a slow growth of renal oncocytoma.

Copy number variation (CNV) indicated several chromosomal losses (Supplementary Figure 2, Supplementary Table 4), similar to a previous report [16]. These include four male patients losing their X chromosome, while 2 out of 6 patient cases partially lost chromosome 1. Patient case 2 featured the highest number of losses on chromosomes 1, 14, 19, 21, and a partial loss of chromosome 7. The sex chromosome loss suggests an involvement of pseudo autosomal regions, which have been the prime candidates for harboring tumor suppressors [20].

We have identified recurring somatic mutations compared to the previous report on an American cohort [16] (Supplementary Figure 3). Insertions in the gene CRIPAK (c.76_77insCA p.S26fs; c.323_324insCA p.L108fs) and single nucleotide variation (SNV) mutations in the gene DRD5 (p.T297P) were identified in both European and American cohorts. As a negative regulator of PAK1 (p21-activated protein kinase 1), CRIPAK is hormone sensitive and was shown to have deleterious mutations in squamous cell- and adenocarcinoma [21] and was further confirmed in other cancer types (COSMIC: COSG57208). These recurring somatic mutations in European and American cohorts may provide potential candidates for the identification of renal oncocytoma diagnostic biomarkers.

**Figure 1:**
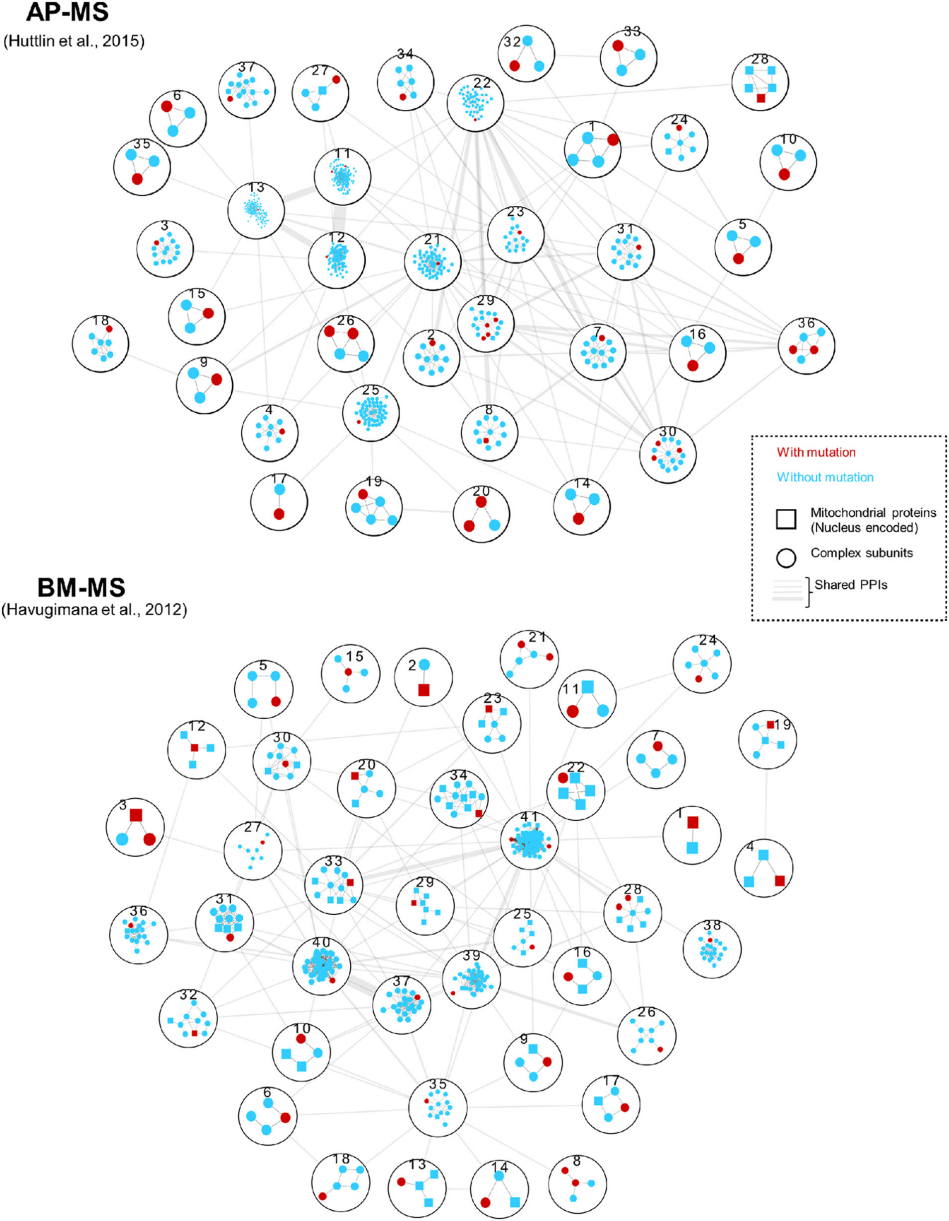
Genes with renal oncocytoma somatic mutations were mapped to previously published protein complexes identified by two biochemical techniques: AP-MS (Affinity-Purification Mass Spectrometry) [24] and BM-MS (Bio Macromolecular-Mass Spectrometry) [22] Protein complexes with non-silent mutations, including missense and frame-shift mutations were numbered and described in Supplementary Table 5. Within each complex, genes with renal oncocytoma somatic mutations were denoted in red, and nucleus encoded mitochondrial proteins were marked with squares. The thickness of edges between protein complexes indicate number of inter protein complex PPIs.

To gain further insight on how mutated genes affecting the renal oncocytoma converge at the protein complex level, we employed a network based approach by mapping subunits of the human protein complexes that were previously reported [22] or predicted using Markov Clustering algorithm [23] from large-scale protein-protein interactions conducted in human embryonic kidney 293 cells [24] to the somatic mutations (excluding 799 silent mutations from the total 1597 nuclear mutations identified) occurring in renal oncocytomas (Figure 1; Supplementary Table 5). In total, we were able to identify 227 renal oncocytoma-associated mutations in 78 distinct human protein complexes, encompassing 182 genes encoding for mitochondrial, and 840 non-mitochondrial proteins. Among the putative complexes compiled, 37 complex subunits with mutations relevant to renal oncocytomas were currently functionally unannotated in CORUM (a manually curated repository of experimentally characterized human protein complexes), whereas the remaining macromolecular assemblies includes, for example, those implicated in cellular metabolism (e.g. Artemis/DNA-dependent protein kinase- [25], pyruvate dehydrogenase-, succinyl-CoA synthetase complexes, and oncocytoma genesis (e.g. missense mutations in DCLRE1C and frame shift mutations in PRKAB1).

The gene *artemis* (DCLRE1C) is one of the subunits of Artemis/DNA-dependent protein kinase complex, whose mutations have been shown to cause hypersensitivity to DNA double-strand breaks [25], and interacts with protein kinase AMP-activated non-catalytic subunit Δ1 PRKAB1 [24]. We have identified missense mutations in DCLRE1C and frame shift mutations in PRKAB1 from renal oncocytoma patient samples. Consistent with the previous studies [16], AMP-activated protein kinase (AMPK) plays crucial roles in oncocytoma genesis, and this complex along with the mutation in its non-catalytic subunit PRKAB1 could be involved in the progression of renal oncocytoma, as it governs the catabolic state upon stress by switching off many ATP-consuming processes.

### Identification of pathogenic low level heteroplasmic mtDNA mutations in renal oncocytomas

**Figure 2:**
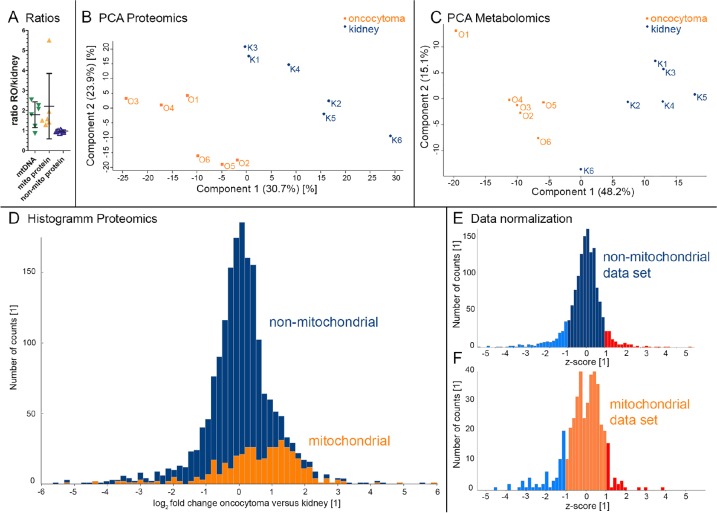
Evaluation of metabolome and proteome profiles by PCA and the distribution of mitochondrial- and non-mitochondrial proteins in renal oncocytomas (RO) versus controls Oncocytomas have a higher mtDNA content, as calculated from WES data (mean 1.79 ± 0.64 SD), a higher amount of mitochondrial protein abundances, (mean 2.22 ± 1.63 SD), and an unchanged non-mitochondrial protein abundance (mean 0.97 ± 0.06 SD) **(A)**. Renal oncocytomas (O, orange, n=6) and kidney (K, blue, n=6) specimen are spatially separated on **(B)** the proteome profile and **(C)** the metabolome profile. The histogram **(D)** (70 bins) shows the number of protein counts versus the log_2_ fold changes of proteins comparing oncocytoma versus kidney samples. Proteins located in the mitochondria are indicated in orange (Human Mito Carta, 1158 entries), whereas non-mitochondrial proteins are shown in blue. A total of 1823 proteins are displayed. A normalized histogram is shown for non-mitochondrial **(E)** and mitochondrial proteins **(F)**. Exceeding's of one STD are indicated in blue and red for proteins considered down - and up-regulated, respectively.

Since oncocytomas with mtDNA mutations feature respiratory defects [4], we examined the assembly of mitochondrial WES reads and found adequate coverage and quality for a reliable mtDNA reconstruction and variant calling (Supplementary Table 6). Comparison of mitochondrial WES reads between renal oncocytomas and healthy kidneys detected a 1.6-fold increase of mtDNA in renal oncocytomas, fitting well to our proteomics data (Figure 2A) with a 2.2-fold increase of proteins localized to mitochondria.

Comparison of healthy kidney tissues and matched renal oncocytomas also identified potentially inherited and tumor-specific mtDNA mutations. In total, 54 germline mutation events shared between matched healthy-tumor pairs recognized by the revised Cambridge Reference Sequence (NC_012920.1) [26], the Reconstructed Sapiens Reference Sequence [27], and by the Macro Haplogroup Consensus Sequence [28] (Supplementary Table 6, Germline_mutations). The Heteroplasmic Fractions (HFs) of germline mtDNA mutations were concordant between tumor and normal tissues, suggesting these events did not undergo changes during tumor development, following cell transformation. Among the 54 germline mutation events, 32 (59.26%) haplogroup defining variants were not considered in the subsequent annotation steps, whereas with the 22 remaining variants, 6 were non-synonymous substitutions (36.4%) (Supplementary Table 6, GM_NonSyn), 8 synonymous (27.2%), and 8 (36.4%) non-protein coding class (Supplementary Table 6, GM_noncodingprotein). However, when considering the disease score and nucleotide variability thresholds [28], only 1 (7874A>G/*MT-CO2)* out of 6 non-synonymous variants were potentially pathogenic (Supplementary Table 6, GM_NonSyn sheet). This homoplasmic mutation, neither contributed in defining other haplogroups nor was annotated in MitoMAP [26], implying that it was novel, and had very low nucleotide variability (0.00031) as estimated in the HmtDB database [29] and in the tracks of *MSeqDR* Mitochondrial Disease pathogenic mutation variant [30].

In the case of 8 variants with non-protein-coding events, 7 mapped in the *MT-DLOOP* region (Supplementary Table 6, GM_noncodingproteins), which is a hot spot for mtDNA alterations as it contains two hypervariable regions (HV1 at positions 16024–16383 and HV2 at positions 57–372), likely of little functional impact. The other variant mapped within the *tRNA-Ala* locus, i.e. the m.5628T>C highlighted in yellow in the GM_noncodingproteins sheet. This variant was considered as damaging by RNA pathogenicity scores provided by MToolBox, a scoring system normalized to a 0-1 range, derived from the literature and different databases. In this case, the variant m.5628T>C had a RNA prediction score equal to 0.65, greater than the fixed pathogenicity threshold 0.35 [31].

Additional examination of renal oncocytomas detected a total of 195 tumor-specific mutations (Supplementary Table 6, Somatic mutations) based on the three mitochondrial reference sets used (indicated above) and did not contribute to define haplogroups. Nearly one quarter (∼17.9%, 35) of the 195 were synonymous, two-thirds (32.3%, 63) non-synonymous substitutions, and less than 10% (19) were non-sense mutations, causing a stop/gain mutation (Supplementary Table 6, SM_stopgain). Among the non-synonymous variants, most of them (54 out of 63; 85.7%) showed a disease score higher than threshold. Of these, 49 (77.8%) had a nucleotide variability lower than the threshold cut-off of 0.026 defined by Santorsola et al. (26) (Supplementary Table 6, SM_NonSyn). Notably, among these, only one mutation was homoplasmic, namely the m.14568C>A/*MT-ND6* (Supplementary Table 6, SM_NonSyn highlighted in green). All the other somatic non-synonymous variants had very low HF values (range between 0.006 and 0.069). Concerning the stop/gain mutations, 8 occurred in CI subunit encoding genes (one in *MT-ND2* and 7 in *MT-ND5*), 8 in the cytochrome B (*MT-CYB)*, 2 in cytochrome c oxidase subunits (one in *MT-CO1* and one in *MT-CO3*) and one in complex V (*MT-ATP6*) (Supplementary Table 6, SM_stopgain). Strikingly, none of the stop/gain mutations was homoplasmic and the HF values did not exceed 0.063.

Furthermore, nearly half of the non-protein coding alterations (78 of 195; 40%) were found in renal oncocytomas, of which two-thirds (52 out of 78) mapped within the regulatory *MT-DLOOP* region. Of these, 48 variants did not define other haplogroups and mostly occurred in conserved sites featuring very low nucleotide variability, ranging from 0 to 0.05 (Pesole and Saccone, 2001) (Supplementary Table 6, SM_noncodingproteins). Moreover, three subjects harbored a mutation in tRNA genes, namely the m.4428G>A/*MT-TM*, m.1611G>A/*MT-TV* and the m.15991C>T/*MT-TP*, showing null nucleotide variability. Noteworthy, the mutation m.15991C>T in tRNA-Pro (*MT-TP*) in the anticodon region of the secondary structure of the tRNA displayed a significant HF of 0.57 (Supplementary Table 6, SM_noncodingproteins).

Ribosomal RNA genes were also found to harbor 22 substitutions. Ten of them were in the *MT-RNR2* and 12 in the *MT-RNR1* gene. By taking into account the PhyloP [32] and PhastCons [33] RNA conservation scores, only 6 rRNA variants resulted to be phylogenetically more conserved than the others (Supplementary Table 6, SM_noncodingproteins). While no homoplasmic tumor specific mtDNA frame shift mutations in protein coding genes were observed, as reported previously [4, 5], a number of distinct pathogenic low level heteroplasmic mutations with cumulative effect was identified, leading to disassembled CI. Interestingly, no mutations in nuclear encoded CI subunits, composed of 46 proteins, were identified, hinting to mtDNA specific feature for renal oncocytomas.

### Renal oncocytomas show increased mitochondrial mass distinct from healthy tissues

To elucidate the changes in the relative abundance of the proteome between renal oncocytomas and matched adjacent healthy kidney tissues, we employed a shotgun liquid-chromatography (LC)-MS/MS framework and identified a total of 6,097 human proteins and quantified nearly half (43%, 2,633) of them (Supplementary Table 7). The reproducibility of the biological replicates was tested by Pearson correlation and visualized in a multi-scatter plot (Supplementary Figure 4). The Pearson correlation (*r* = 0.7-0.9) was consistent between replicates, indicating the robustness of the data quality. Application of stringent cut-off (values present in at least 3 out of 6 samples in each renal oncocytoma patient tissue group) resulted in 1823 quantified proteins relative to tissues from healthy subject controls. Among those proteins identified in renal oncocytomas, 141 showed significant differences in protein abundance (67 up- and 74 down- regulated; Supplementary Table 8A). In order to calculate ratios for the highest regulated proteins where the protein abundance was below the detection limit in one group, we replaced these missing values from normal distribution, resulting in 1462 protein groups (Supplementary Table 8B).

The proteome profiling further revealed renal oncocytomas and healthy adjacent kidney tissues as distinct groups in a principal component analysis (Figure 2B). Statistical analysis revealed a non-Gaussian distribution of the proteome. A shift to increased mitochondrial protein amount was observed, the hallmark of renal oncocytomas (Figure 2D). A normalized histogram is shown for non-mitochondrial (Figure 2E) and mitochondrial proteins (Figure 2F) individually. Based on the total abundance of mitochondrial and non-mitochondrial proteins, we calculated a 2.2-fold increase of mitochondrial proteins, in line with the 1.8-fold increase in mtDNA amount identified in our WES data (Figure 2A). Also, the protein abundance for proteins affected by CNV loss was significantly lower compared to random values (p= 0.011, Supplementary Figure 5), suggesting a proportional relation between gene dose and the protein abundance.

### Renal oncocytomas feature a significant increase of metabolic processes localized in mitochondria, with the exception of CI activity

We then employed a gene set enrichment analysis (GSEA) [34] to assess, if a prior defined sets of proteins show statistically significant, concordant differences between renal oncocytomas and healthy kidney tissues. GSEA revealed four significantly up- and five significantly down-regulated KEGG and Reactome pathways in renal oncocytomas versus controls (*p* ≤0.01 and FDR ≤ 0.15; Table 1, Supplementary Table 9). These include mitochondria related pathways, such as the TCA cycle, pyruvate metabolism, and protein import that were significantly up-regulated in renal oncocytomas (Table 1). Since GSEA recognizes pathways regulated in one direction to be significant, the respiratory chain with CI regulated in the opposite way compared to all other OXPHOS complexes was therefore not significantly altered in renal oncocytoma.

**Table 1:** Significant pathways of proteome data by Gene set enrichment analysis (GSEA): Collapsedlist of significantly regulated KEGG and Reactome pathways of oncocytoma and kidney samples

Up-regulated pathway in renal oncocytoma	Size	NES	NOM p-value	FDR q-value
Mitochondrial protein import	27	2.24	<0.0001	0.00
TCA cycle	17	1.80	0.0020	0.04
Pyruvate metabolism	8	1.72	0.0020	0.11
TCA cycle and respiratory electron transport	66	1.69	<0.0001	0.10
**Down-regulated pathway in renal oncocytoma**				
Pentose phosphate pathway	8	−1.89	<0.0001	0.08
Cell cell communication	17	−1.84	0.0019	0.11
Fructose and mannose metabolism	7	−1.80	<0.0001	0.11
Glycine serine and threonine metabolism	6	−1.80	<0.0001	0.09
Biological oxidations	13	−1.76	0.0040	0.14

List was manually reduced based on multiple matching of same gene sets, the entire list is shown in Supplementary Table 9. Size, number of proteins in the pathway; NES, normalized enrichment score.

**Figure 3:**
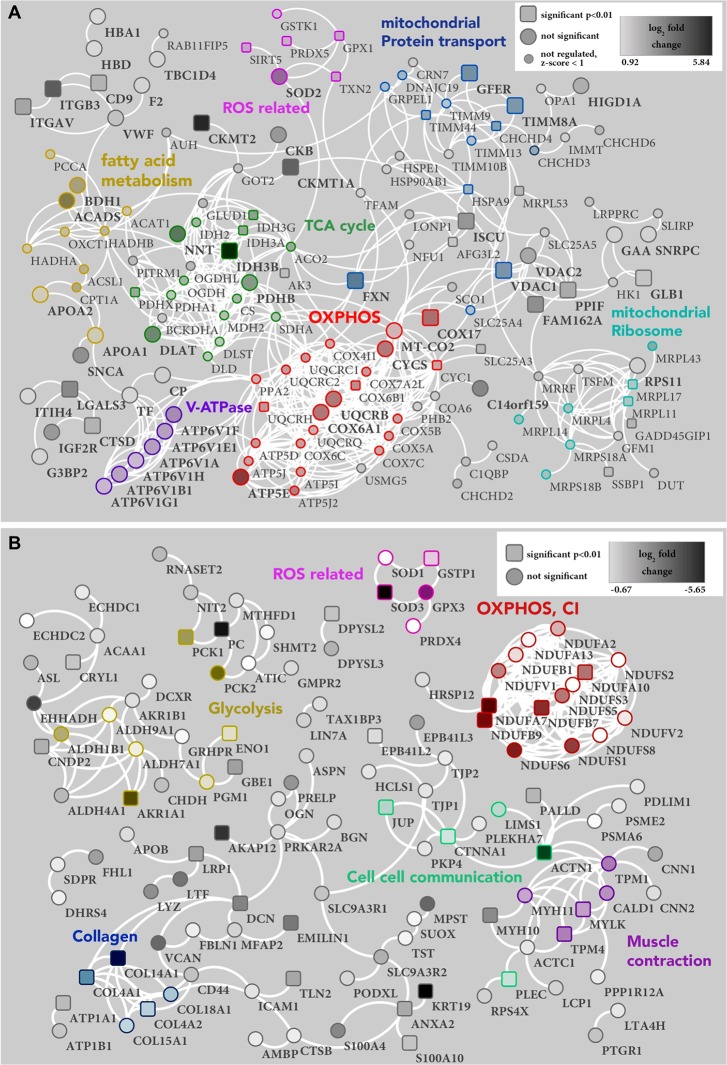
PPI network of proteins **(A)** up- and **(B)** down-regulated in renal oncocytoma. (A) The PPI network of 238 proteins with a high confidence level (0.7), which exceeded one STD from the median, shows 151, proteins with at least one connection (white lines). Unconnected entries have been removed. Significant (p-value < 0.01) protein differences are represented as a square node, others as a circle shape. Small nodes indicate proteins with a z-score < 1 in the mitochondrial data set (Figure 2E), which were therefore not considered as regulated. The density of each protein node resembles the log_2_ fold change of oncocytoma versus kidney tissue, ranging from very light (0.92) to very dark (5.84). Identified clusters are indicated in color. Reactome pathways: mitochondrial protein transport (blue), Fatty acid metabolism (ochre), TCA cycle (green) and OXPHOS (red). Manual cluster: mitochondrial ribosome (turquoise), ROS related proteins (pink) and V-ATPase (purple). (B) The PPI network of 222 proteins with a high confidence level (0.7), which exceeded one STD from the median, shows 122, proteins with at least one connection (white lines). Unconnected entries have been removed. Significant (p-value < 0.01) protein differences are represented as a square node, others as a circle shape. The density of each protein node resembles the log_2_ fold change of oncocytoma versus kidney tissue, ranging from very light (−0.67) to very dark (−5.65). Identified clusters are indicated in color. Reactome pathways: cell cell communication (green), muscle contraction (purple) and OXPHOS (red). KEGG pathway: glycolysis (ochre). Manual cluster: ROS related proteins (pink) and collagen (blue).

A PPI network of up-regulated proteins, defined by one standard deviation (SD) of the mean was created using the online platform http://string-db.org [35], and displayed once more the huge impact on mitochondrial protein abundances, such as proteins involved in the 55S mitochondrial ribosome, TCA cycle, fatty acid metabolism, mitochondrial protein transport, and respiratory chain (Figure 3A), except CI (Figure 3B) in renal oncocytomas. To elucidate which mitochondrial proteins were regulated, the data were split into two sets, mitochondrial and non-mitochondrial proteins. Proteins were only defined as differentially regulated if exceeding a value of one SD from the mean within each data set. Mitochondrial proteins were therefore regarded as up-regulated if the log_2_ fold change was >2.041 and down-regulated if the change was <-0.6267. For non-mitochondrial proteins, cut-offs were set to 0.9226 and −0.9868 for up- and down-regulation, respectively. These identified proteins were highlighted in a PPI network figure as up- (Figure 3A) and down-regulation (Figure 3B).

**Figure 4:**
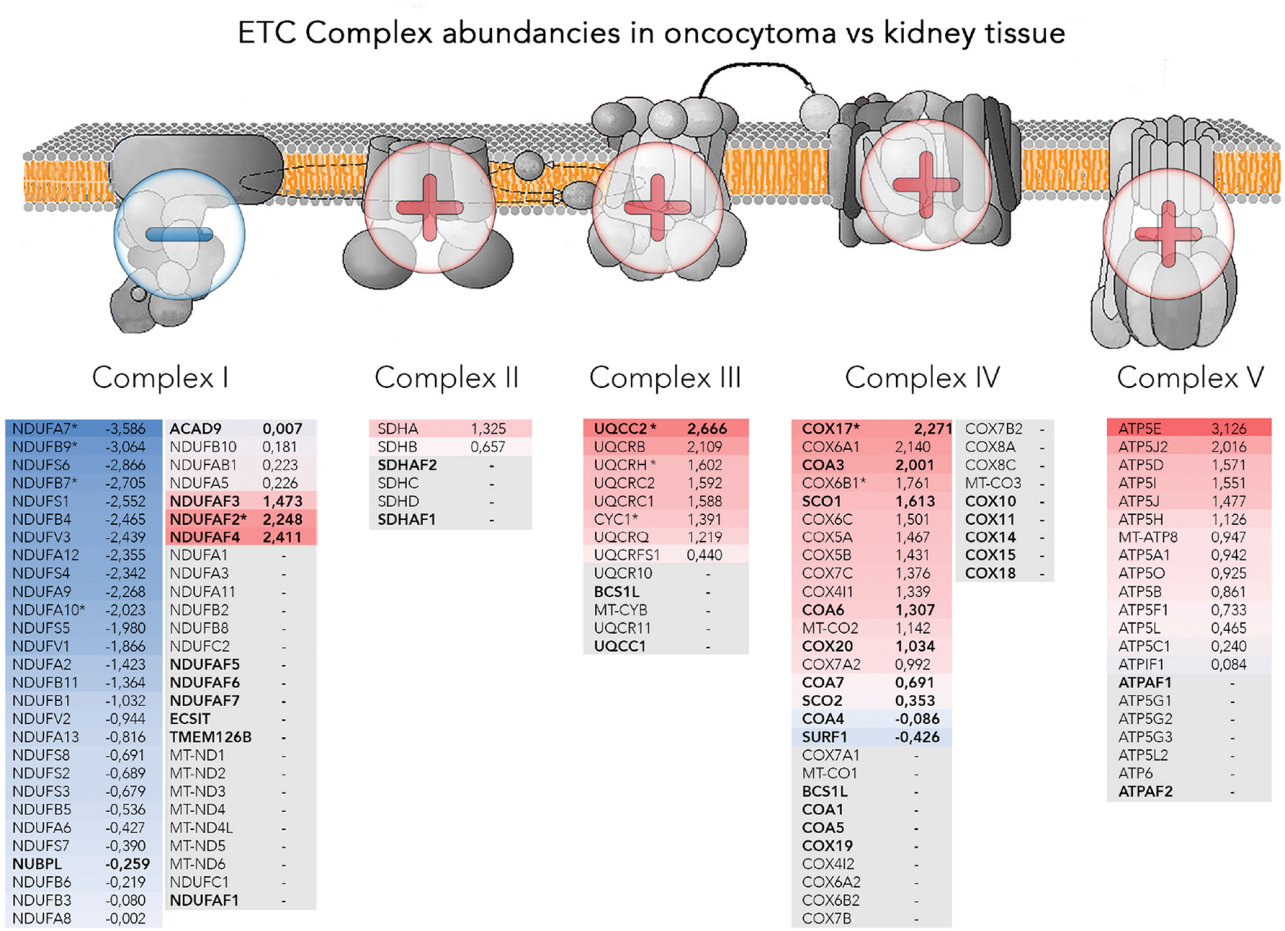
Abundancies of the OXPHOS protein complexes Illustrated are schemes of the four OXPHOS complexes and the ATPase, embedded in the mitochondrial membrane with additional lists of identified subunits and assembly factors and the according log_2_ fold change between renal oncocytoma versus kidney samples. The color gradient intensity in the subunit expresses the low (blue) or high (red) abundance of this protein in renal oncocytoma. The operators overlaying the OXPHOS indicate complexes II, III, IV and the ATPase being generally high abundant, whereas subunits of complex I are found in reduced amounts. Bold written genes refer to assembly factors, not part of the final complex, ^*^ are significantly regulated proteins. The OXPHOS scheme was adapted from the Kyoto Encyclopedia of Genes and Genomes (KEGG).

A detailed analysis of OXPHOS complexes revealed a significant down-regulation of CI subunits and up-regulation of all other complexes and the ATPase (Figure 4), in agreement with our previously detected enzyme activities in renal oncocytomas [4]. In particular, all identified assembly proteins were up-regulated, including those of complex I (Figure 4), likely indicating an ongoing compensatory effort in oncocytic cells.

### Up-regulation of anti-mitochondrial ROS defense in renal oncocytomas

Proteins involved in ROS detoxification were up- as well as down-regulated. For example, mitochondria-localized glutathione peroxidase 1 (GPX1, 3-fold) and superoxide dismutase 2 (SOD2, 6-fold) were up-regulated in renal oncocytomas, whereas cytoplasmic glutathione S-transferase P (GSTP1, 2.3-fold), glutathione peroxidase 3 (GPX3, 8-fold), superoxide dismutase (SOD1, 2-fold), and extracellular superoxide dismutase (SOD3, 35-fold) were down-regulated (Figure 3A and 3B, Supplementary Table 8). These results suggest the occurrence of a ROS-mediated stress within mitochondria due to a respiratory dysfunction, and a consequent increase of detoxifying enzymes within the specific subcellular compartment. Furthermore, we identified a gap in the gamma-glutamyl cycle, a pathway for the synthesis and degradation of glutathione. All identified enzymes were basically unchanged, except gamma-glutamyl transpeptidase 1 and 5 (GGT1, 50-fold; GGT5, 15-fold), which were significantly down-regulated in renal oncocytomas. Betaine-homocysteine methyltransferase (BHMT, 200-fold), an enzyme that catalyze the remethylation from homocysteine to generate methionine was significantly decreased. Typically, homocysteine do not contribute to the conversion of dimethylglycine and methionine, instead, they can be used to fuel GSH synthesis [36, 37]. Thus, the breakdown of GSH is interrupted and the synthesis of GSH is augmented.

### Renal oncocytomas decrease their glycolytic capacity

An interesting question was how renal oncocytomas regulate their glycolytic capacity in the light of compromised CI. GSEA revealed significant down-regulated pathways such as the pentose phosphate pathway, fructose and mannose metabolism, glycine, serine and threonine metabolism, biological oxidations, and cell communications (Table 1, Supplementary Table 9). Fructose-1, 6-bisphosphatase (FBP1), a rate-limiting enzyme in gluconeogenesis was 200-fold down-regulated in renal oncocytoma. FBP1 was shown to exhibit dual tumor-suppressive functions, in gluconeogenesis as well as a HIF1A inhibitor. An intriguing regulatory relationship between FBP1 and hypoxic responses was shown in ccRCC to oppose carcinoma progression [17]. Consistent with this, fructose-bisphosphate aldolase B (ALDOB, 275-fold) and sorbitol dehydrogenase (SORD, 32-fold) were down-regulated. The highest down-regulated enzymes of the “glycine serine and threonine metabolism” were: phosphoglycerate dehydrogenase (PHGDH, 22-fold), serine hydroxymethyltransferase 1 (SHMT1, 60-fold), betaine-homocysteine methyltransferase (BHMT, 200-fold), glycine aminotransferase (GATM, 300-fold), and agmatinase (AGMAT, 175-fold). The down-regulation of these enzymes might explain the significant lower amount of several amino acids in renal oncocytomas.

Additionally, the glycolytic enzyme alpha-enolase (ENO1, 2-fold) which produces phosphoenolpyruvate was decreased in renal oncocytomas. Pyruvate carboxylase (PC, 40-fold), which transfers a carboxyl group to pyruvate to form oxaloacetate (OAA) and phosphoenol pyruvate carboxykinase (PCK1, 5-fold and PCK2, 9-fold), which catalyze the conversion of OAA to phosphoenolpyruvate (PEP), the rate-limiting step in the metabolic pathway that produces glucose from lactate were two of the most decreased enzymes abundant in renal oncocytomas. Thus, the metabolic flux shunts away from the TCA cycle, resulting in glycolysis and gluconeogenesis to entirely stall.

The pathway reductive carboxylation of glutamine-derived α-ketoglutarate for de novo lipogenesis is active in most cell lines and depends on cytosolic isocitrate dehydrogenase 1 (IDH1) [38]. We found IDH1 to be 25-fold decreased, whereas the mitochondrial encoded enzymes were all increased, IDH3B, 20-fold; IDH3G, 6-fold; IDH3A, 3-fold; IDH2, 3-fold. This indicates that reductive carboxylation of glutamine does not play a role as energy source in renal oncocytomas. Likewise, transketolase (TKT) and transaldolase (TALDO) are two major enzymes for the non-oxidative branch of pentose phosphate pathway (PPP). In rapidly proliferating cancer cells, the non-oxidative PPP can be accelerated to meet the increased needs of ribonucleotides through elevated expressions of TKT and TALDO [39]. We found both enzymes not to be regulated, indicating that the level of ribonucleotides in renal oncocytoma is constant, which could be a barrier for the progression of renal oncocytomas.

Mitochondrial creatine kinase S-type (CKMT2, 57- fold) and U-type (CKMT1A, 18-fold) are responsible for the energy and metabolite transfer between mitochondria and cytoplasm (i.e. the creatine phosphate shuttle), and were increased in renal oncocytomas. Both are reversibly catalyzing the transfer of phosphate between ATP and various phosphogens, thus playing a central role in energy transduction in tissues with large, fluctuating energy demands. Their up-regulation can be a sign of imbalanced energy supply between mitochondria and cytosol. Most cancer cells predominantly produce energy by a high rate of glycolysis rather than by OXPHOS, known as the Warburg effect [40]. Thus, an opposite Warburg effect, as detected here, seems to be a beneficial feature of renal oncocytomas.

### Renal oncocytomas have increased levels of V-ATPases, proteins involved in Ca^2++^ homeostasis and fewer cytoskeletal proteins

Vacuolar-type H^+^-ATPases (V-ATPase) acidify a wide array of intracellular organelles and pump via ATP hydrolysis protons across intracellular and plasma membranes. Six V-ATPases were found to be (2.5-fold) up-regulated in renal oncocytomas (Figure 3A). Acidity is one of the main features of tumors and V-ATPases control the microenvironment by proton extrusion to the extracellular medium [41].

The pathways “cell-cell communication” and “muscle contraction” were significantly down-regulated in renal oncocytomas (p-value ≤ 0.05; Table 1), consisting of protein members such as myosin, tropomyosin, collagens, vinculin, talin, caldesmon, and vimentin, the latter serving as a marker to differentiate renal oncocytomas from renal cell carcinoma [42]. For example, alpha-actinin-1 (ACT1), a F-actin cross-linking protein was 15-fold reduced, junction plakoglobin (JUP) which influences the arrangement and function of both the cytoskeleton and the cells within the tissue was 3-fold decreased. The pathway “glycosaminoglycan metabolism” plays a structural role in connective tissue, cartilage, bone, and blood vessels [43] and was decreased as well. The lack of a strong cytoskeleton and connective intracellular proteins fits well to the squashy like texture of oncocytomas. Furthermore, cytoskeleton alterations were linked previously to mtDNA mutations and OXPHOS status [44].

Significant up-regulation of calcium signaling proteins were identified in renal oncocytomas. Calcium signaling by 1-phosphatidylinositol 4,5-bisphosphate phosphodiesterase gamma-2 (PLCG2, 45-fold), producing the secondary messenger inositol 1,4,5-trisphosphate (IP3) that release calcium ions from the ER into the cytosol activates stromal interaction molecule 1 (STIM1, 5-fold), resulting in an influx of extracellular calcium ions [45–47]. While the intracellular Ca^2+^ homeostasis is frequently altered in cancer cells [48], the consequences in renal oncocytomas still have to be elucidated.

### Renal oncocytomas display an altered metabolome to stress response, amino acids, and “energy carrier” levels

**Figure 5:**
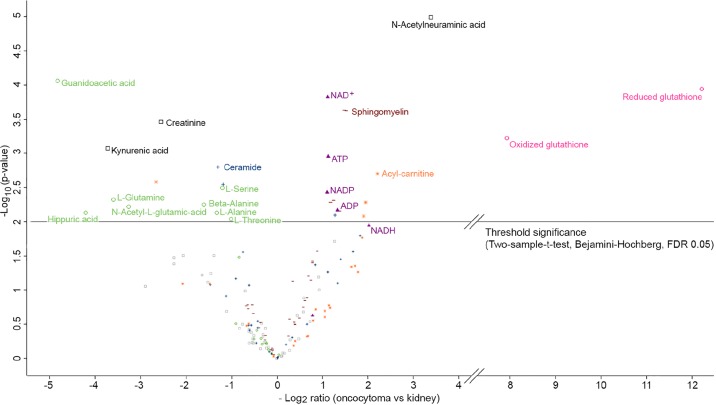
Volcano plot of log2 abundance ratios of oncocytoma versus kidney tissues against the -log10 (p-value) of the metabolome Indicated are following metabolites: ROS scavengers in red (circle); OXPHOS related metabolites in purple (filled triangle); amino acids and intermediates of their pathways in green (circle); acyl-carnitines in orange (star); sphingomyelins in brown (dash); ceramides in blue (cross) and others in black (rectangle). Metabolites above the horizontal line are significant after considering the Benjamini-Hochberg correction (FDR of 0.05) for multiple testing and are additionally listed in Table 2.

To quantify the relative difference in metabolite changes and to elucidate key metabolic switches between renal oncocytomas and patient matched kidney tissues, we applied a targeted LC-MS/MS based on multiple reaction monitoring (MRM). In total, 159 metabolites were relatively quantified (Supplementary Table 10). The Pearson correlation coefficients were highly similar, ranging from 0.845 to 0.977 in oncocytomas and 0.839 to 0.977 in kidney tissues (Supplementary Figure 4), suggesting overall consistency in the quality of metabolite data sets. Statistical analysis by a two sample *t*-test with Benjamini-Hochberg (BH, FDR of 0.05) correction for multiple testing revealed 29 significantly (p-value <0.01) regulated metabolites (13 down, 16 up) in oncocytomas vs. kidney tissues (Figure 5; Table 2).

**Table 2:** List of significantly regulated metabolites between renal oncocytomas and healthy kidney tissues

metabolite	t-test p-value	Fold change oncocytoma vs kidney	Group
Reduced glutathione	0.0001	5413	amino acids, peptides, and analogues
Oxidized glutathione	0.0005	267	amino acids, peptides, and analogues
N-Acetylneuraminic acid	<0.0001	10.44	sugar acids and derivatives
Glutaryl-carnitine	0.0020	4.64	acyl-carnitines
Cervonyl-carnitine	0.0052	3.87	acyl-carnitines
Arachidyl-carnitine	0.0083	3.76	acyl-carnitines
Sphingomyelin (d16:1/24:1(15Z))	0.0002	2.88	phosphosphingolipids
Sphingomyelin (d18:0/22:0)	0.0002	2.79	phosphosphingolipids
Sphingomyelin (d18:1/26:0)	0.0070	2.60	phosphosphingolipids
ADP	0.0074	2.53	nucleotides
Ceramide (d18:1/14:0)	0.0080	2.43	ceramides
Sphingomyelin (d18:1/22:0)	0.0049	2.37	phosphosphingolipids
Sphingomyelin (d18:1/26:1(17Z))	0.0052	2.26	phosphosphingolipids
ATP	0.0012	2.19	nucleotides
NAD^+^	0.0002	2.18	nucleotides
NADP	0.0041	2.16	nucleotides
L-Threonine	0.0090	−2.02	amino acids, peptides, and analogues
Ceramide (d18:1/26:1)	0.0029	−2.28	ceramides
L-Serine	0.0032	−2.32	amino acids, peptides, and analogues
Ceramide (d18:1/25:1)	0.0016	−2.46	ceramides
L-Alanine	0.0075	−2.51	amino acids, peptides, and analogues
Beta-Alanine	0.0055	−3.08	amino acids, peptides, and analogues
Creatinine	0.0003	−5.91	imidazolines
Isovaleryl-carnitine	0.0026	−6.34	acyl-carnitines
N-Acetyl-L-glutamic acid	0.0060	−9.62	amino acids, peptides, and analogues
L-Glutamine	0.0047	−12.22	amino acids, peptides, and analogues
Kynurenic acid	0.0008	−13.27	quinoline carboxylic acids
Hippuric acid	0.0074	−18.53	benzamides
Guanidoacetic acid	0.0001	−28.46	amino acids, peptides, and analogues

Metabolites with p-values ≤ 0.01 after Benjamini-Hochberg correction for multiple testing with a FDR of 0.05, were regarded as significant.

As with our proteomics analysis (Figure 2B), the metabolite profiling revealed renal oncocytomas and healthy adjacent kidney tissues as distinct groups in a principal component analysis (Figure 2C). The most striking difference was found for the ROS scavenger reduced glutathione (GSH, >5000-fold in renal oncocytomas) and its oxidized version glutathione disulfide (GSSG, >250-fold; Figure 5, Table 2). GSH levels were very low in several normal kidney tissue samples then background levels were taken in to account to build a ratio. With respect to the defective respiratory electron chain, the metabolites entering or exiting the OXPHOS system, such as NAD^+^, NADP, ADP and ATP were significantly (*p*-value <0.01, 2-fold) more abundant in oncocytomas. NADH (Benjamini-Hochberg corrected *p*- value of 0.012) was 4-fold increased. Consistent with non-functional CI, lacking the ability to oxidize NADH, we saw increased levels of NADH in renal oncocytomas.

Several amino acids and intermediates were significantly less abundant in renal oncocytomas (p ≤ 0.05), including glutamine, serine, alanine, Δ-alanine, threonine, N-acetyl-glutamic acid and guanidoacetic acid. The sugars sucrose (p-value = 0.0766) and trehalose (p-value = 0.0605) were not significantly deregulated, but were both 3-fold less abundant in oncocytomas. However, the level of lactic acid, the endpoint of anaerobic breakdown of glucose, was unchanged. Five sphingomyelins and three acyl-carnitines of different chain lengths were significantly (p ≤ 0.05) up-regulated in oncocytomas (Table 2). The metabolite with the lowest p-value (0.00001) was N-acetylneuraminic acid with a 10-fold increase, commonly known as sialic acid and regarded as a tumor marker. Thus, our screen revealed distinct metabolite profiles between benign oncocytomas and healthy kidney tissues and identified an increase in OXPHOS metabolites and high levels of glutathione in renal oncocytomas.

### Computational assessment of metabolic processes indicated a low metabolism in renal oncocytomas

In order to identify differences in metabolic fluxes between kidney and renal oncocytomas, we applied a kinetic model to unravel the functional implications of protein abundance changes of metabolic enzymes. This model was originally created to monitor changes in the liver metabolism (personnel communication). Three different blood glucose levels were considered for the simulations: low, normal, and high glucose levels (4 mM, 7.36 mM, and 10 mM).

**Figure 6:**
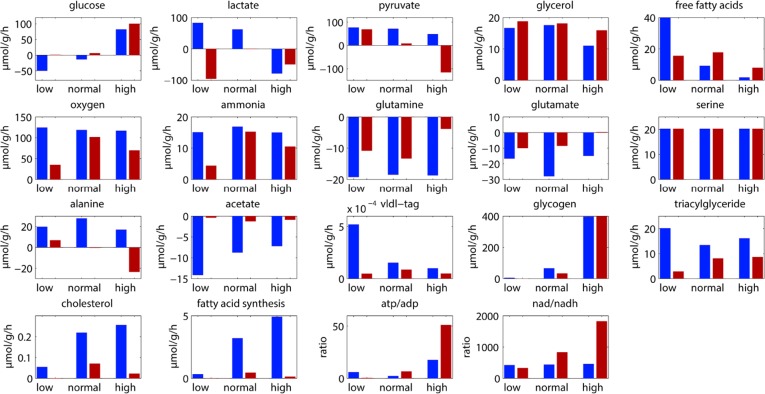
Simulated metabolic levels at three different glucose concentrations (low, 4 mM; normal, 7. 36 mM; high, 10 mM) in healthy kidney (blue) and renal oncocytomas (red)

The simulations revealed remarkable differences between the metabolic profiles of kidney and renal oncocytomas (Figure 6). The lipid metabolism was reduced in renal oncocytomas (e.g. lower rates of fatty acid synthesis, cholesterol synthesis, triglycerides). Free fatty acids uptake was decreased at low glucose conditions and increased at high glucose conditions. Δ-oxidation was almost absent as NADH cannot be oxidized in CI. This lead to a severe energy depletion (ATP/ADP ratio) of the tumor under fasting conditions. The oxygen consumption rate was decreased at low glucose conditions, but almost normal at high glucose conditions. In these conditions, CI was bypassed and electrons were directly transferred to ubichinon by the glycerol-phosphate shuttle. The tumor was more glycolytic than the kidney, but this was not a consequence of increased glycolysis, but rather due to severely decreased gluconeogenesis. At high glucose concentration when gluconeogenesis was not operating, glycolytic rates were comparable. Glycogen was slightly reduced, less glutamate and glutamine were exported in renal oncocytomas.

Altogether, these simulations revealed that renal oncocytomas have higher ATP and NADH levels under normal glucose levels, consistent with our metabolomics data. This is achieved by a severe reduction of ATP consuming processes, rather than an increased energy metabolism in renal oncocytomas.

## DISCUSSION

In this study, we specifically asked which molecular pathways are reprogrammed in renal oncocytomas to sustain growth and prevent progression to malignant forms. The main characteristics of renal oncocytomas are mutations within the respiratory chain, especially in CI [4, 6]. Despite the general shift to higher mitochondrial protein abundance in renal oncocytoma, nearly all CI subunits showed a significant (p ≤ 0.05) lower abundance in our data set. Interestingly, assembly factors of all OXPHOS complexes, were found to be up-regulated, including those of CI. Consistent with this, genes involved in the OXPHOS and CI were shown to be up-regulated in the transcriptome of 11 renal oncocytomas [16]. MtDNA mutations in complex I genes were the cause of a failed assembly, and as a consequence the enzymatic activity was lost [4]. Crucial core subunits were entirely missing, as shown by single up to 100% homoplasmic frame shift mutations, leading to short truncated subunits [4]. As well, multiple low level pathogenic heteroplasmic mtDNA mutations resulted in a dramatic decrease of CI protein abundancies, leading to incorrectly assembled or non-functional complex, which may become highly unstable and degrade fast, as previously shown in oncocytic cells bearing ND1 mutations [4, 5].

Surprisingly, the abundance of glycolytic enzymes or metabolites was not increased as one might suspect. Such a phenomenon was observed previously with ADP/ATP translocase 1 (SLC25A4 −/−) [49], an enzyme which exports mitochondrial ATP to the cytosol in exchange for ADP. SLC25A4 −/− mice featured a dramatic proliferation of mitochondria and increased abundance of genes involved in OXPHOS, at the same time, a decrease of glycolytic genes. This was explained by the hyperoxic state, as O_2_ is not consumed and cytosolic ATP decreased, resulting in uncontrolled proliferation of mitochondria to compensate for the cytosolic ATP deficiency [50]. An induction of mitochondrial OXPHOS should be associated with the reciprocal down-regulation of glycolysis, which was observed in SLC25A4−/− [49] as well as in our study. In agreement with this, CI-deficient cells acquiring oncocytic phenotype in mouse xenografts were shown to be incapable of gaining a Warburg-like glycolytic profile, despite their severe respiratory damage, in association with a chronic HIF1a destabilization, whereby down-regulation of glycolytic genes occurs [51].

Furthermore, HIF1A can inhibit mitochondrial biogenesis via peroxisome proliferator-activated receptor gamma coactivator 1-Δ (PPARGC1B) [52]. As oncocytic tumors have less HIF1A, this can explain the increased number of mitochondria in oncocytomas. One of the key enzymes in gluconeogenesis and hypoxia, FBP1, was the dramatic reduction in ccRCC [17], which we found as well to oppose tumor progression. Increased creatine kinases can be a sign of imbalanced energy supply between compartments, but as “energy shuttle”, they do not recruit new energy sources for renal oncocytoma either. Alternatively, Golgi disassembly to block trafficking and secretion to conserve energy resulting in a defective autophagy and increased defective mitochondrial mass is discussed as a potential cause of renal oncocytoma [16]. However, no indications of altered autophagy or Golgi pathways were found in our proteome survey. An impairment of the respiratory chain due to mtDNA mutations was recently shown to alter the cytoskeleton structure, accompanied by mis-orientation of the Golgi body [44].

We identified several V-ATPases to be increased in renal oncocytomas; they hydrolyze ATP for the regulation of protons being pumped from the cytoplasm into the lumen of intracellular acidic compartments and play a key role in urinary acidification [53]. As renal oncocytomas are not fulfilling any kidney function, one explanation could be a role in lactic acid removal to avoid cytosolic acidosis. Most notably, our metabolome data show for the first time that renal oncocytomas do not appear to suffer from an “energy crisis”. Instead, they feature even higher levels of metabolites such as NAD^+^, NADH and ADP and ATP. As we measured whole cell lysates, we cannot distinguish between cytosolic and organellar metabolite concentrations, but the latter have shown to differ considerably between compartments [54]. Therefore, a lower ATP concentration solely in the mitochondrial matrix is plausible (as indicated by creatine kinases), even if the total concentration was higher. The metabolome comparison of human renal oncocytomas and patient-matched healthy kidney tissues revealed a striking up-regulation of the antioxidant glutathione, leading to the hypothesis that oncocytomas depend on normal ROS levels to sustain survival and growth. Glutathione prevents ROS damage to important cellular components by donating reducing equivalents for free radicals, peroxides, lipid peroxides and heavy metals [55], as well as maintenance of the intracellular redox balance and the essential thiol status of proteins [56]. Indeed, measurement of free glutathione can be used to evaluate the redox and detoxification status of cells in relation to its protective role against ROS. The high antioxidant level can therefore be linked to the high production of ROS by deficient CI or CIII. In support of our hypothesis, ROS levels in thyroid oncocytoma derived XTC.UC1 cells showed no alterations. This was explained by the differential expression of ROS detoxifying enzymes [15, 57]. In this regard, the gamma-glutamyltranspeptidase 1 and 5 (GGT1, GGT5), and the betaine homocysteine S-methyltransferase (BHMT), contributing to intracellular GSH and homocysteine maintenance were significantly down-regulated in renal oncocytomas, indicating a degradation brake in the gamma-glutamyl cycle, which might be the molecular reason for the high GSH levels. These observations may also support the hypothesis that oncocytic cells must keep low levels of ROS as they appear to have impaired mitophagy/autophagy, as previously observed [58]. If ROS damages were to become consistent, there would be no way of eliminating damaged cell components, ultimately leading to cell death. It is therefore plausible that in the ever-changing cancer population, oncocytic cells with a higher detoxifying capacity be positively selected. At least in yeast, high levels of GSH have been shown to prevent mitophagy, not related to its scavenging properties, but rather to the fueling effect of the glutathione pool [59, 60].

**Figure 7:**
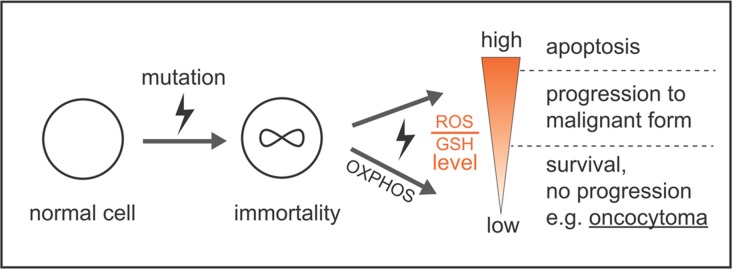
Model by which ROS and GSH levels govern a stable balance sustaining survival and growth, avoiding high mutation rates and apoptosis, which prevents malignant tumor progression and cell death One mutation for uncontrolled proliferation and a second mutation specifically affecting the OXPHOS system are necessary for the unique features of oncocytomas. High ROS levels by a defect CI or CIII are instantly eliminated by high GSH levels to avoid any further damage to the cell, which supports stable, but uncontrolled growth.

It was proposed that at least two distinct mutations are necessary for oncocytic tumor development. One leads to uncontrolled proliferation and the second to specific impairment of the OXPHOS system [61]. It remained unclear why progression to malignant forms of cancers is not observed, especially regarding the high mutagenic potential of an elevated ROS production. We conclude that excess levels of mitochondrial-derived radicals are instantly decomposed by GSH, resulting in normal ROS levels, which prevent any further malignant transformations in oncocytomas, but promote survival and growth (Figure 7), along with the lack of HIF1a stabilization and a stall of glycolytic pathways, which would contribute to increased mitochondrial mass (via PPARGC1B) and prevent malignancy. But it still remains a chicken or egg causality dilemma, if mtDNA mutations were the cause or the consequence of high ROS levels. It was shown previously in cybride cells that only specific mtDNA mutations, such as LOHN mutations increase ROS, thus there is a direct link between mtDNA mutations and ROS levels [62].

Comparing our GSH levels to ccRCC, less dramatic elevated levels were found. In malignant ccRCC, glutathione levels were 2.6 to 2.8-fold increased [63], up to 20% elevated in RCC [64], about 5 to 7-fold raised in another RCC study [65], and 145-fold in ccRCC [17]. Increased GSH levels have also been shown to be directly correlated with cellular proliferation and metastatic activity [66]. Hence, a balance of ROS and antioxidant levels is key factor for tumor survival and growth.

Several clinical approaches are aiming to manipulate GSH levels in several tumor types to reduce or even stop the survival and proliferation advantages, e.g. inducing apoptosis. For example, irradiation is more effective on cancer cell lines containing low GSH levels [67] and drug resistance correlates with increased GSH levels in ovarian cancer [68] and RCC [69]. The therapeutic strategy to reduce GSH levels in cancer is hampered by the fact that GSH depletion leads to up-regulation of antioxidant genes, as shown by the transcription activator NFE2L2 in response to oxidative stress [70]. Other ROS scavenging agents for therapeutic purposes have also been investigated (reviews: [71–73]).

Additionally, the NAD^+^/NADH balance impacts the shift from respiration to glycolysis in cancer [8]. NADH is a well-known inhibitor of several TCA cycle enzymes, such as pyruvate dehydrogenase complex (PDC), citrate synthase, and oxoglutarate dehydrogenase complex (OGDC). Therefore, 4-fold elevated NADH level observed is likely to hinder this metabolic pathway. A number of studies have demonstrated that malignant transformation, which is not the case in renal oncocytomas, is associated with an increase in glycolytic flux and anaerobic cellular lactate excretion [74]. Our observed down-regulation of detected amino acids including the pathway intermediates, such as guanidoacetic acid, hippuric acid, and N-acetyl-L-glutamic acid indicates that the entire amino acid pathways were down-regulated. On one hand, amino acids are utilized to attenuate oxidative stress. This has been shown for glutamine in RCC and goes along with the up-regulation of glutathione, made of glycine, cysteine, and glutamine [65]. On the other hand, they serve as substrates to fuel anaerobic ATP synthesis.

One of the most important carbon and nitrogen sources for a number of cancers is glutamine [75]. The conversion of glutamine and serine to lactate via glutaminolysis and serinolysis complements glycolysis. Reductive glutamine metabolism was shown to be highly dependent on cytosolic isocitrate dehydrogenase-1 (IDH1) [38]. As IDH1 was significantly reduced and metabolic simulation indicated that the lipid metabolism was stalled in renal oncocytomas, it is unlikely that glutamine will be converted via citrate to lipids during reductive carboxylation and this alternative pathway can therefore be excluded to be a main energy source. Macropinocytosis-mediated internalization of extracellular protein and its subsequent intracellular degradation, for example, was shown as a mechanism for amino acid supply in Ras-transformed cancer cells [76]. Another correlation to the speed of cancer-cell division is glycine consumption. Fast replicating tumors are consumers, slowly dividing cells even release small amounts of glycine. Glycine metabolism may therefore represent a metabolic vulnerability in rapidly proliferating cancer cells [77]. Furthermore the serine synthesis pathway was shown to be essential in breast cancer [78], and murine embryonic stem cell proliferation has a dependence on threonine catabolism [79]. Hippuric acid, an important metabolite of the phenylalanine metabolism, was found to be the most down-regulated (35 -fold) metabolite in RCC [80], correlating well with our 18-fold decrease in renal oncocytomas. We initially assumed that anaerobic pathways such as glycolysis, glutaminolysis and serinolysis will be up-regulated, as the only alternative to compensate for the defective OXPHOS system. The down-regulation of amino acids as well as the sugars (e.g. sucrose and trehalose) indicates that they are indeed metabolized. However, we detected significantly increased metabolites of the energy machinery, for example, ATP, NADH and NADP and unchanged levels of lactic acid. Lactate dehydrogenase B levels were even decreased in renal oncocytomas [81]. The lack of elevated lactic acid levels can, at least in part, be explained by its instant secretion into the plasma by our detected increased abundance of V-ATPases in renal oncocytomas, where it serves as standard tumor marker for cancer and in the acidification of the environment, adding to the consistency and architecture of oncocytomas. Our simulations also revealed a severe reduction of ATP consuming processes. Thus, the need for activating alternate energy pathways is not required. Most important questions such as how much biomass is build up by renal oncocytomas, and at what proportion oncocytomas use up the energy compared to normal metabolic functions still remains unclear.

Additional metabolites, like increased sialic acid (N-acetylneuraminic acid), a tumor marker for most cancer cells matched well to 10-fold up-regulation in renal oncocytomas. Also consistent with our results, acyl-carnitines found to be increased in RCC in all grades [65] as well as in human urine [82]. It was suggested that the elevated acyl-carnitine levels were utilized by other processes not directly related to energy metabolism, as the majority of fatty acid β-oxidation enzymes were down-regulated in proportion to RCC grade [65].

Whether renal oncocytomas can progress to more aggressive forms, such as the chromophobe RCC type is still an unanswered question. There are no literature reports indicating such events, except one reporting a metastasis originating from renal oncocytomas [83]. Thus, it seems to be a very rare event, supporting our hypothesis of a stable balance between ROS and GSH levels to sustain survival and growth.

## EXPERIMENTAL PROCEDURES

### Tissue dissection and verification of renal oncocytoma

Six nephrectomy specimens were collected in liquid nitrogen immediately after surgery and preserved at −80°C. From the collected tissue samples, frozen histologic sections were performed, and stained with hematoxylin and eosin. The diagnosis of renal oncocytoma and the corresponding matched tumor-free kidney tissue were made under consideration of macroscopic and histologic features according to the WHO classification criteria. If necessary, immunohistochemistry with at least staining of CK7, CK20 and S100A1 were performed. Only cases with a clear diagnosis of an oncocytoma were regarded for the study.

### Exome sequencing

DNA was isolated from remaining pellets from the metabolite extraction using a DNA purification kit following the manufacture's protocol (QIAmp DNA Mini Kit for Tissues, QIAGEN, Hilden, Germany). In brief, samples were digested by proteinase K at 56°C, overnight and RNAse A treated at 70°C, before subjection for exome sequencing.

The library preparation was performed according to Agilent's SureSelect protocol (SureSelectXT Human All Exon V5, protocol version B4 August 2015) for Illumina paired end sequencing. In brief, 200 ng of genomic DNA (in 50 μl low TE) were sheared for 6^*^60 sec on a Covaris™ S2 (duty factor 10%, intensity 5, 200 cycles per burst).

The fragmented DNA (150-200 bp) was purified using AMPure XP beads and subjected to an end-repair reaction. Following another purification step the DNA was 3′adenylated and furthermore purified. Paired-end adaptors were ligated and the afterwards purified library was amplified with 10 amplification cycles. The amplified library was purified, quantified and hybridized to the probe library for exome capturing. Captured fragments were purified using streptavidin-coated beads and eluted with 30 μl NFW. Using Herculase-enzyme, the enriched libraries were amplified and indexed with barcoded primers followed by cleanup and quantification.

The resulting libraries were pooled and subjected to Illumina NextSeq500 paired end sequencing (6 libraries/FC; 2 × 150 bp) and yielded about 120 million reads (150 bp) per sample.

Quantification of the SureSelect captured library: Before sequencing, the samples were re-quantified with two methods. First, the size and concentration was checked on the Agilent 2100 Bioanalyzer and in a second step the enrichment efficiency was estimated by qPCR (Applied Biosystems) using a primer set for an enriched exon (fw: ATCCCGGTTGTTCTTCTGTG and rv: TTCTGGCTCTGCTGTAGGAAG) and a primerset in an intron region as a negative control (fw: AGGTTTGCTGAGGAACCTTGA and rv: ACCGAAACATCCTGGCTACAG). In general, the CT-values of target and control fragments differed by 6 to 10, thus confirming a very good enrichment of our target regions.

After diluting the captured libraries to 10 nM, Genome Analyzer single read flow cells were prepared on the supplied Illumina cluster station and 36 bp single end reads on the Illumina Genome Analyzer IIx platform were generated following the manufacturer's protocol. Images from the instrument were processed using the manufacturer's software to generate FASTQ sequence files.

### Bioinformatic mt and nuclear DNA analysis

The FASTQ files were used as input of the MToolBox pipeline [84], in order to extract mitochondrial DNA sequences and quantify each variant allele heteroplasmy and related confidence interval. The same pipeline allows haplogroup prediction of mtDNA sequences, detection of mismatches, insertions and deletions and the functional annotation of the identified variants already implemented in MToolBox [84]. The in *silico* prioritization criteria here adopted and published in Santorsola et al. (2016) [28] are intended to easily target the mitochondrial DNA variants of clinical interest, by prioritizing those variants recognized against the mitochondrial reference sequences (rCRS, RSRS and MHCS), which occurred in non haplogroup-defining sites, featuring nucleotide variability lower than nucleotide variability cutoff (0.0026) and having a disease score above the disease score threshold (0.43, 0.35 and 0.60 for non synonimous coding for proteins, tRNA and rRNA variants respectively).

The analysis-ready bam files were prepared based on the guidelines suggested by the “best practice pipeline” [85, 86]. Briefly, the raw reads were aligned to the human reference genome (GRCh37) with bwa algorithms. The PCR duplicates were marked and removed by Picard software (https://broadinstitute.github.io/picard/). The base recalibration and indel realignment were processed using the GATK [87] (Version 3.6) package. Somatic point mutations were called with Vardict [88] algorithms. Copy Number Variations (CNVs) were detected with CNVkit [89]. The exome sequencing data analysis was implemented by bcbio_nextgen (https://github.com/chapmanb/bcbio-nextgen) pipeline. The annotation of the somatic mutations was implemented using ANNOVAR [90].

### Sample preparation for proteomics

About 10 mg frozen tissue per sample was homogenized under denaturing conditions with a FastPrep (three times for 60 s, 6.5 m x s^−1^) in a buffer containing 4% SDS, 0.1 M DTT, 0.1 M Tris pH 7.8, followed by sonication for 10 min, boiled at 95°C for 5 min and precipitated with acetone at −20°C overnight. Lyophilized proteins were dissolved in 6 M GdmCL, 10 mM TCEP, 40 mM CAA, and 100 mM Tris pH 8.5. Samples were boiled for 5 min at 95°C and sonicated for 15 min in a water sonicator. The lysates were diluted 1:10 with nine times volume of 10% ACN and 25 mM Tris, 8.5 pH, followed by a trypsin digestion (1:100) at 37°C overnight. Subsequent, the peptides were purified with C18 columns and further fractionated by strong cation exchange (SCX) chromatography. Five μg of each SCX fraction and a non-fractioned sample were used for proteome profiling and analyzed by MaxQuant (v1.5.3.30).

### LC-MS instrument settings for shotgun proteome profiling and data analysis

LC−MS/MS was carried out by nanoflow reverse phase liquid chromatography (Dionex Ultimate 3000, Thermo Scientific, Waltham, MA) coupled online to a Q-Exactive HF Orbitrap mass spectrometer (Thermo Scientific, Waltham, MA). Briefly, the LC separation was performed using a PicoFrit analytical column (75 μm ID × 55 cm long, 15 μm Tip ID (New Objectives, Woburn, MA) in-house packed with 3-μm C18 resin (Reprosil-AQ Pur, Dr. Maisch, Ammerbuch-Entringen, Germany). Peptides were eluted using a gradient from 3.8 to 40% solvent B in solvent A over 100 min at 266 nL per minute flow rate. Solvent A was 0.1% formic acid and solvent B was 79.9% acetonitrile, 20% H_2_O, 0.1% formic acid). Nanoelectrospray was generated by applying 3.5 kV. A cycle of one full Fourier transformation scan mass spectrum (300−1750 m/z, resolution of 60,000 at m/z 200, AGC target 1e^6^) was followed by 12 data-dependent MS/MS scans (resolution of 30,000, AGC target 5e^5^) with a normalized collision energy of 25 eV. In order to avoid repeated sequencing of the same peptides a dynamic exclusion window of 15 sec was used. In addition, only the peptide charge states between two to eight were sequenced.

Raw MS data were processed with MaxQuant software (v1.5.3.30) [91] with the Andromeda search engine [92], and the UniProtKB with 70,228 entries, released in 02/2016. A false discovery rate (FDR) of 0.01 for proteins and peptides, a minimum peptide length of 7 amino acids, a mass tolerance of 4.5 ppm for precursor and 20 ppm for fragment ions were required. A minimum Andromeda score of 0 and 40 (delta score 0 and 9) for unmodified peptides and modified peptides was applied, respectively. A maximum of two missed cleavages was allowed for the tryptic digest. Cysteine carbamidomethylation was set as fixed modification, while N-terminal acetylation and methionine oxidation were set as variable modifications. The label-free software MaxLFQ [93], integrated in MaxQuant, was used for quantification. MaxQuant processed output files can be found in Supplementary Table 7, showing peptide and protein identification, accession numbers, % sequence coverage of the protein, q-values and LFQ intensities. Contaminants as well as proteins identified by site modification and proteins derived from the reversed part of the decoy database were strictly excluded from further analysis. The mass spectrometry proteomics data have been deposited to the ProteomeXchange Consortium via the Pride [94] partner repository with the dataset identifier PXD007633.

### Metabolite extraction and profiling by targeted LC-MS

About 23-38 mg of six unrelated and frozen renal oncocytoma and corresponding healthy kidney tissues were used for metabolite profiling. Metabolite extraction and tandem LC-MS measurements were done as previously reported by us [95]. In brief, methyl-tert-butyl ester (MTBE), methanol, ammonium acetate and water were used for metabolite extraction. Subsequent separation was performed on a LC instrument (1290 series UHPLC; Agilent, Santa Clara, CA), online coupled to a triple quadrupole hybrid ion trap mass spectrometer QTrap 6500 (Sciex, Foster City, CA), as reported previously [96]. Transition settings for multiple reaction monitoring (MRM) are provided in Supplementary Table 11. The mass spectrometry data have been deposited to the publically available repository PeptideAtlas with the identifier PASS00831. All original LC-MS generated QTrap wiff- files as well as MuliQuant processed peak integration q.session- files can be downloaded via http://www.peptideatlas.org/PASS/PASS00831.

The metabolite identification was based on three levels: (i) the correct retention time, (ii) up to three MRM's (iii) and a matching MRM ion ratio of tuned pure metabolites as a reference [94]. Relative quantification was performed using MultiQuantTM software v.2.1.1 (Sciex, Foster City, CA). The integration setting was a peak splitting factor of 2 and all peaks were reviewed manually. Only the average peak area of the first transition was used for calculations. Normalization was done according to used amounts of tissues and subsequently by internal standards, as indicated in Supplementary Table 11.

### Experimental design and statistical rationale, pathway-, and PPI network analyses

For proteome and metabolome data sets, a two-sample t-test was performed. Multiple test correction was done by Benjamini-Hochberg with a FDR of 0.05 by using Perseus (v1.5.0.8) [97]. Significantly regulated proteins and metabolites were marked by a plus sign in according Supplementary Tables (Supplementary Tables 8 and 10).

For comprehensive proteome data analyses, gene set enrichment analysis (GSEA, v2.2.3) [34] was applied in order to see, if priori defined sets of proteins show statistically significant, concordant differences between renal oncocytoma and kidney tissues. Only proteins with valid values in at least three samples in each tissue group were averaged and used for GSEA analysis and log_2_ transformation (Supplementary Table 8A), or six valid values in at least one group with replacing missing values from normal distribution for the other group (Supplementary Table 8B) to rescue the most regulated proteins. GSEA default settings were used, except the minimum size exclusion was set to 5, KEGG v5.2 and Reactome v5.2 were used as gene set database. The cut off for significantly regulated pathways was set to a p-value ≤ 0.01 and FDR ≤ 0.25. For protein-protein interaction (PPI) network analyses, the software tool String v.10 has been used to visualize networks of significantly down-regulated proteins with a confidence level of 0.7 [35]. Protein nodes which were not integrated into a network were removed.

### Computational assessment of metabolic processes indicated a low metabolism in renal oncocytomas

In order to identify differences in metabolic fluxes between kidney and renal oncocytomas, we applied a mathematical kinetic model of central metabolism to unravel the functional implications of protein abundance changes of metabolic enzymes. This model, comprising central energy metabolism, as well as carbohydrate, lipid and amino acid metabolism, as well as the key electrophysiological processes at the inner mitochondrial membrane including the mitochondrial membrane potential and the generation and utilization of the proton motive force, was originally created to monitor changes in the liver metabolism (Berndt et al. in review). The model takes into account the regulation in the activity of metabolic enzymes through substrate concentration, allosteric effectors, reversible phosphorylation and protein abundances. In order to compare healthy and tumor cells, the experimentally determined protein abundance changes between the two cell types were used to scale the Vmax values of the corresponding enzymes. The defect of complex I was explicitly taken into account by setting the Vmax value to 10% of its normal value, corresponding to the CI subunit with the lowest abundance in renal oncocytoma.

To assess metabolic changes under different physiological conditions, three different blood glucose levels corresponding to low, normal, and high glucose levels (4 mM, 7.36 mM, and 10 mM) were considered for the simulations.

## CONCLUSIONS

In summary, cancer-specific pathogenic mtDNA mutations lead to a decreased CI abundance and defective respiratory chain in renal oncocytomas. The Warburg effect is antagonized by FBP1 and the metabolic switch to glycolytic pathways, PPP, and reductive glutaminolysis cannot be made; thus were dramatically decreased as well. As a consequence, renal oncocytomas reduced ATP consuming pathways to reach sufficient energy supplies. The main metabolic feature and new hallmark of OXPHOS deficient renal oncocytomas is the increase of GSH. Extremely elevated GSH levels counteract high ROS levels originating from defective CI, thereby probably prohibiting apoptosis, tumor transformation and progression to malignant forms, but at the same time, sustaining survival and growth as benign tumor.

## SUPPLEMENTARY MATERIALS FIGURES AND TABLES




















