# Supplementary material for: Renal oncocytoma characterized by the defective complex I of the respiratory chain boosts the synthesis of the ROS scavenger glutathione
Source: Oncotarget. 2017 Nov 11;8(62):105882–904. doi: 10.18632/oncotarget.22413 (PMC5739687; doi:10.18632/oncotarget.22413)
Supplement: Supplementary file 1 [file oncotarget-08-105882-s001.pdf]

Renal oncocytoma characterized by the defective complex I of the respiratory chain boosts the synthesis of the ROS scavenger glutathione

SUPPLEMENTARY MATERIALS

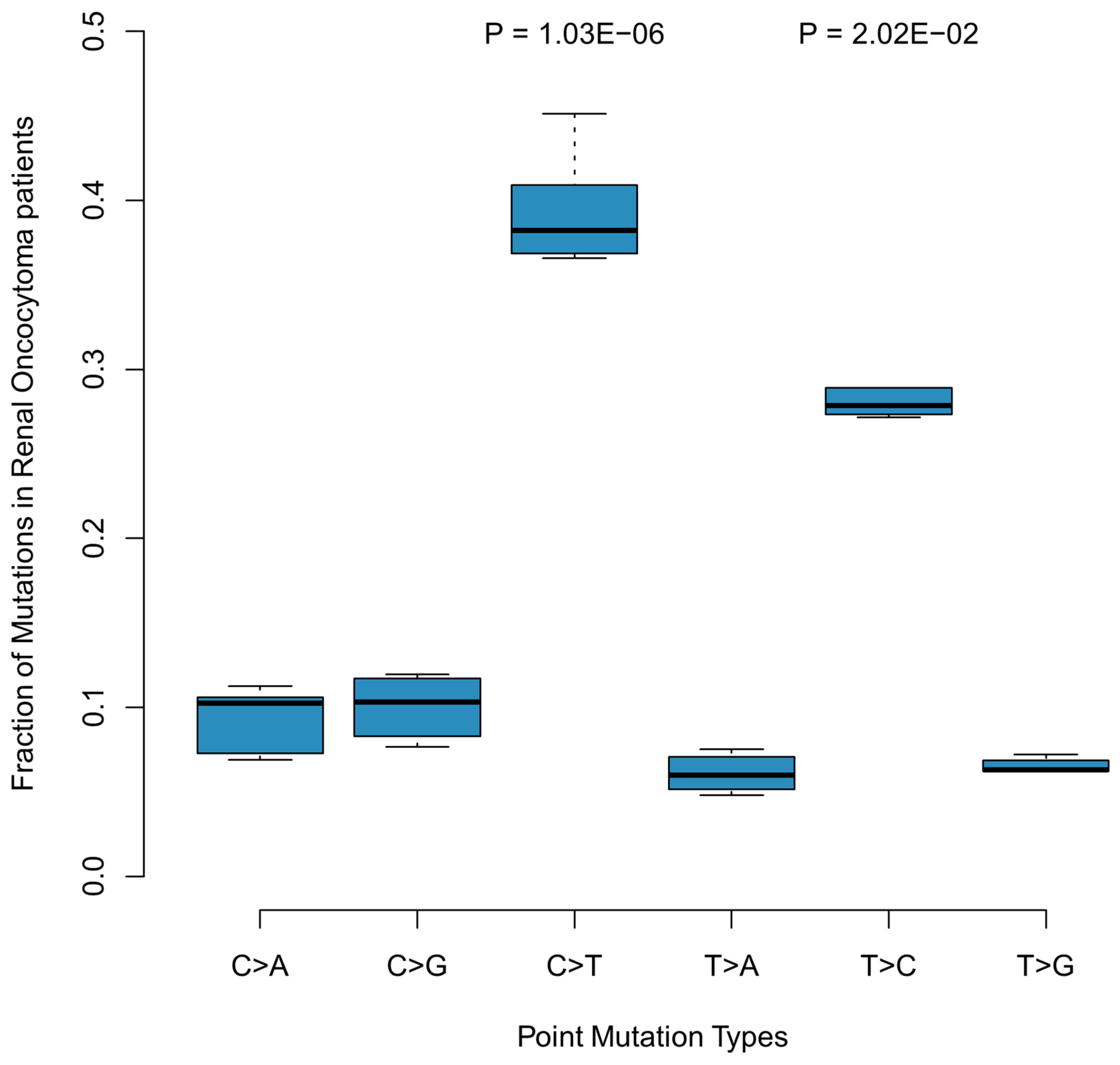

Supplementary Figure 1: Somatic point mutation types identified in renal oncocytoma patients. Statistical significance was derived from Wilcoxon rank-sum test.

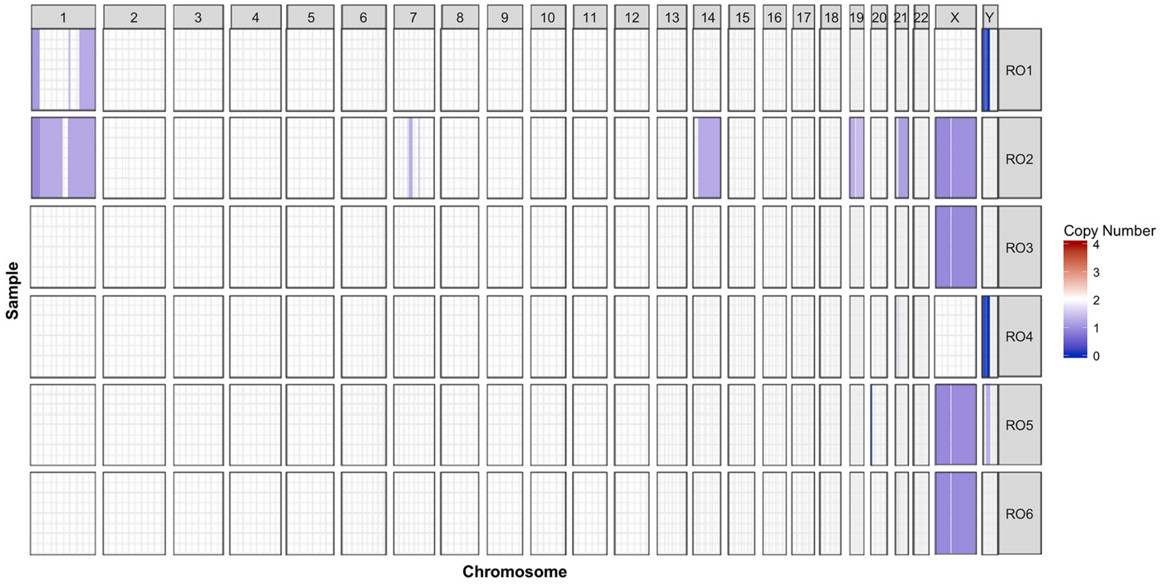

Supplementary Figure 2: Copy number variation (CNV) analysis results from renal oncocytoma patients. Dark blue indicates chromosomal loss, while red indicates chromosomal gain, which was not detected.

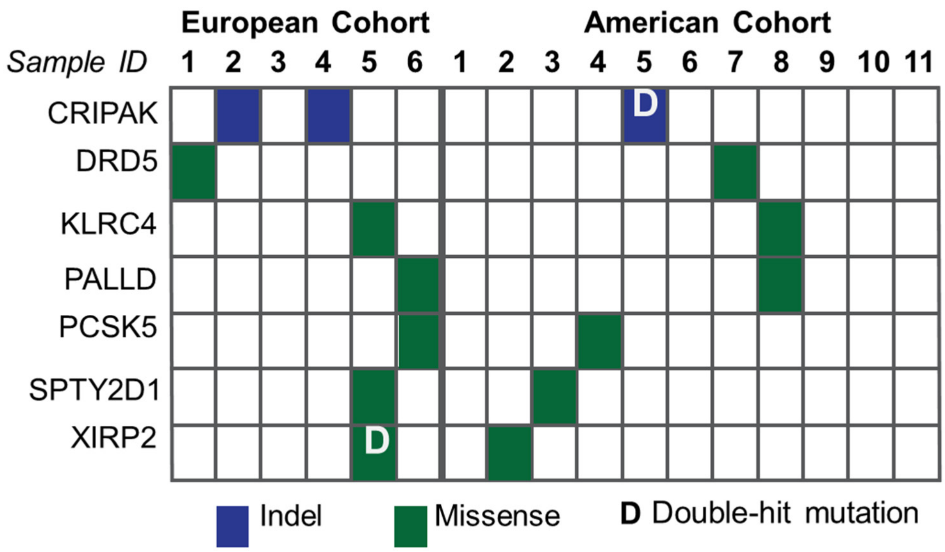

Supplementary Figure 3: Somatic mutations in comparison to American cohort [16]. Only SNVs common between European and American cohorts are shown; the complete list of detected somatic SNVs and indels is reported in Supplementary Table 2.

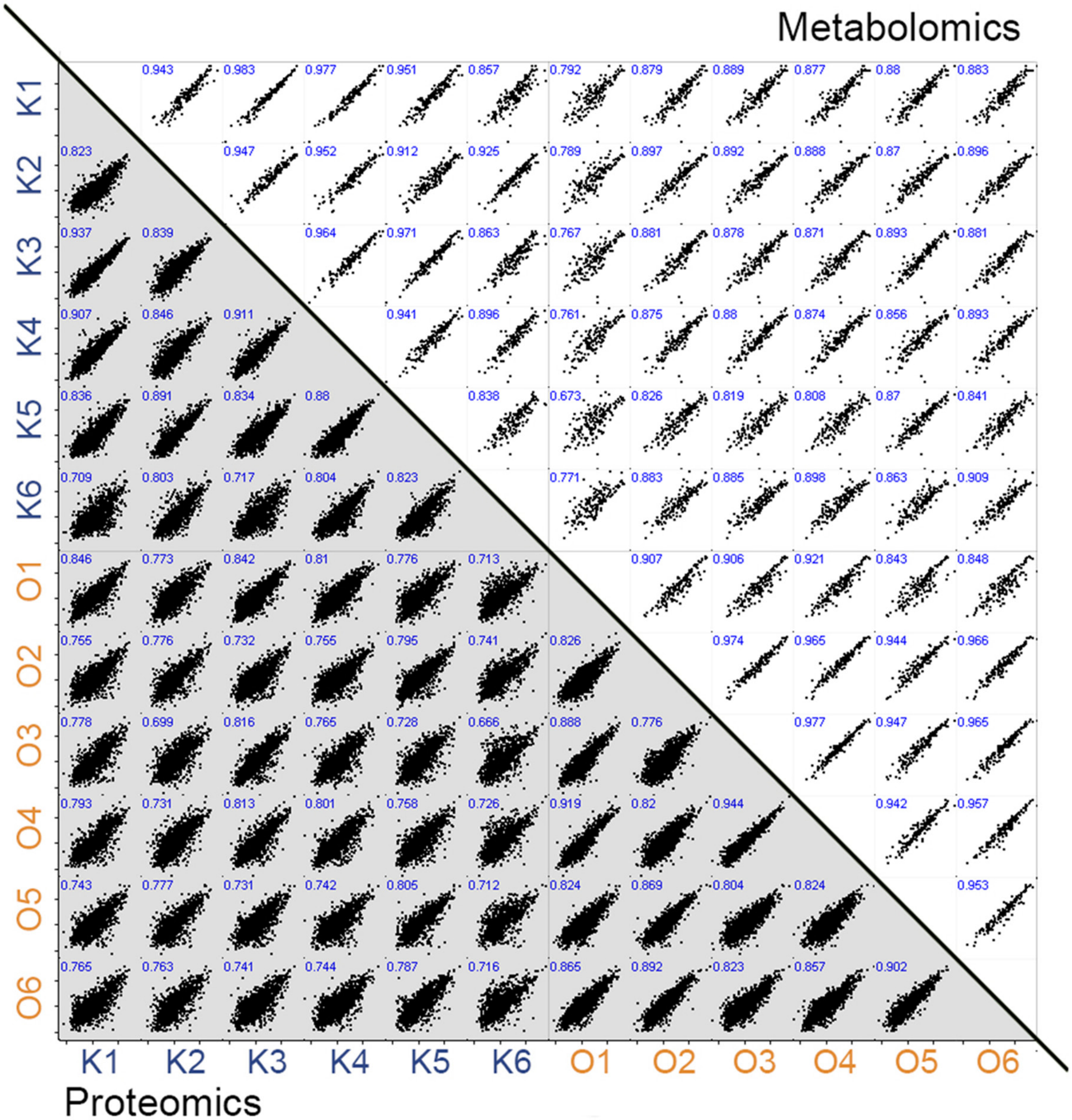

Supplementary Figure 4: Pearson correlation of the proteome and metabolome between all renal oncocytomas and kidney tissues is shown in scatter plots. K = kidney, O = oncocytoma.

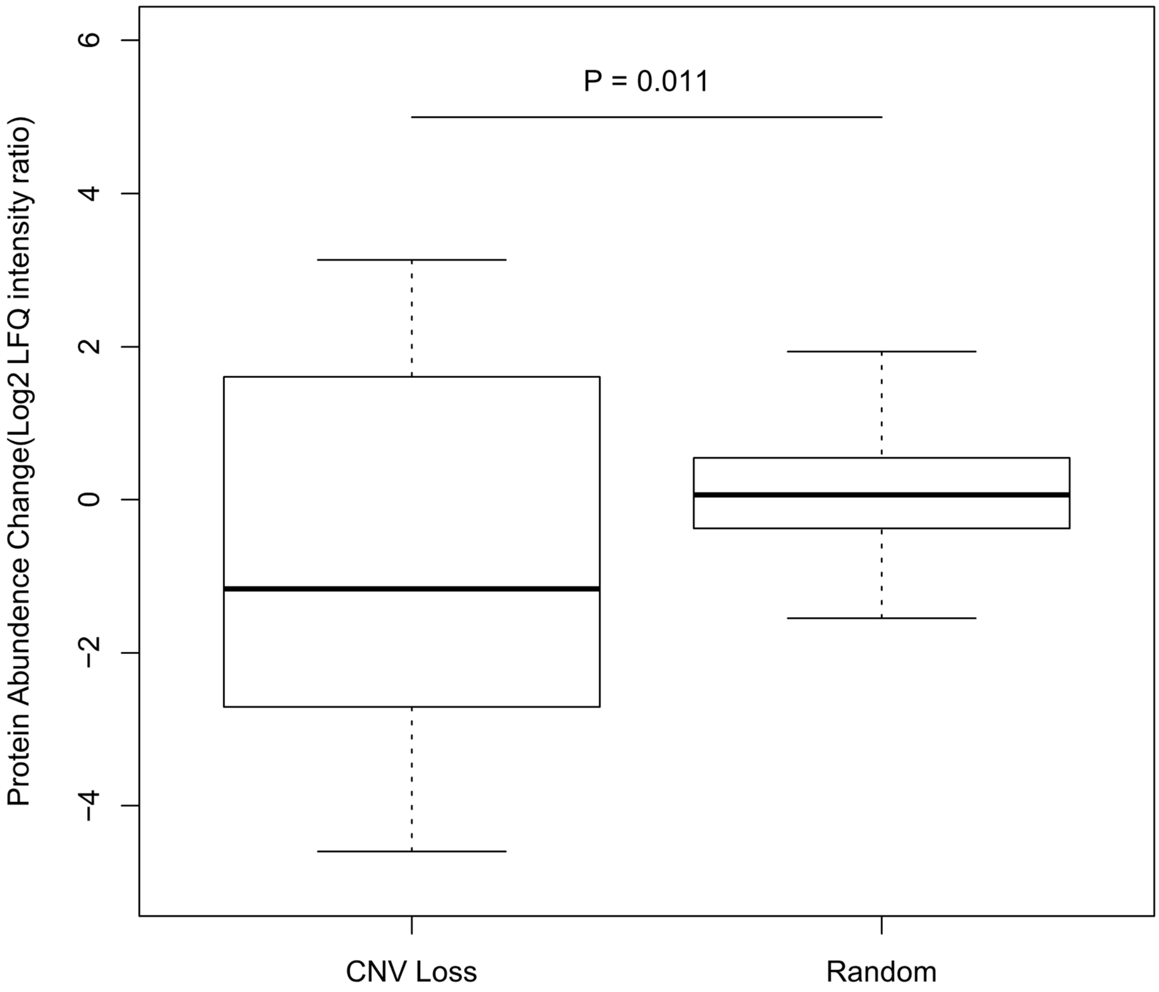

Supplementary Figure 5: Changes of protein abundances of genes with loss of copy number in renal oncocytoma patients compared to random sets. The random sets were generated via bootstrapping proteins were not affected by CNV. Statistical significance was derived from Wilcoxon rank-sum test.

Supplementary Table 1: The percentage of reconstructed genome covered by the assembly, the mean coverage depth, the number of contigs obtained and the best predicted haplogroup are reported for each sample

Sample	mtDNA coverage	Mean read depth	Number of contings	Best predicted haplogroup(s)
O1	100	308.66	1	W3a1d
K1	99.99	252.18	1	W3a1d
O2	99.59	207.61	2	H2a1c
K2	99.95	81.68	1	H2a1c
O3	99.95	173.79	1	H5a1
K3	99.6	83.74	2	H5a1
O4	99.89	206.37	2	U5a1b
K4	99.81	114.75	2	U5a1b
O5	99.63	162.39	2	H7a1a
K5	99.16	71.98	4	H7a1a
O6	99.84	104.92	2	H5a1g
K6	99.99	123.03	1	H5a1g

O = oncocytoma, K = kidney, same sample numbers are derived from the same patient.

Supplementary Table 2: List of all detected somatic mutations in renal oncocytoma patients

See Supplementary File 1

Supplementary Table 3: Numbers of somatic point mutations categorized by mutation types in renal oncocytoma patients

	C>A	C>G	C>T	T>A	T>C	T>G
RO1	62	73	230	47	173	39
RO2	77	68	261	44	187	47
RO3	76	77	264	51	202	52
RO4	78	88	282	41	200	47
RO5	63	70	412	47	264	57
RO6	44	50	247	29	202	32
**Sum**	**400**	**426**	**1696**	**259**	**1228**	**274**

transition (yellow)
transversion (green)

Supplementary Table 4: Complete list of genes with Copy Number Variations

See supplementary File 2

Supplementary Table 5: Complete list of complexes consists of genes with somatic mutations identified in renal oncocytoma samples

See supplementary File 3

Supplementary Table 6: Pairwise comparison of healthy kidney tissue and renal oncocytoma mtDNA mutations obtained by applying the MToolBox package

See supplementary File 4

Supplementary Table 7: MaxQuant output file featuring the proteome profile of renal oncocytoma and matching kidney tissue with LFQ intensities

See supplementary File 5

Supplementary Table 8: List of protein groups and LFQ intensities identified in at least three samples of the kidney and oncocytoma group

See supplementary File 6

Supplementary Table 9: Pathway enrichment analysis (GSEA) of proteins between renal oncocytoma and kidney tissue

See supplementary File 7

Supplementary Table 10: List of identified metabolites (including HMDB and KEGG ID) in oncocytoma and kidney tissues as $\log_2$ transformed peak areas

See supplementary File 8

Supplementary Table 11: Detection parameters for metabolite multiple reaction monitoring (MRM) methods

See supplementary File 9
